# Supplementary material for: Investigating the effect of oblique image acquisition on the accuracy of QSM and a robust tilt correction method
Source: Magn Reson Med. 2022 Dec 8;89(5):1791–808. doi: 10.1002/mrm.29550 (PMC10953050; doi:10.1002/mrm.29550)
Supplement: Supplementary file 1 — Figure S1. Results in the numerical phantom from oblique image volumes tilted about the x‐axis. The volumes were padded to ensure no parts of the original image volume were cut off during rotations. The QSM‐tuned structural similarity index (XSIM) measurements of QSM calculated with tilt corrections before background field removal with projection onto dipole fields (PDF) (A), Laplacian boundary value (LBV) (B), and variable‐kernel sophisticated harmonic artifact reduction for phase data (V‐SHARP) (C) agree with the unpadded results (Figure 5). The XSIM measurements comparing tilt correction schemes before susceptibility calculation with thresholded k‐space division (TKD) (D), iterative Tikhonov regularization (E), and linear weighted linear total variation (TV) (F) methods are also in agreement with the unpadded results (Figure 6, bottom row) Figure S2. Results in the numerical phantom from oblique image volumes tilted about the y‐axis with padded image volumes to ensure no parts of the original image volume were cut off during rotations. The XSIM measurements of QSM calculated with tilt corrections before background field removal with PDF (A), LBV (B), and V‐SHARP (C) agree with the unpadded results and rotations about the x‐axis (Figure 5, Supporting Information Figure S1A–C). The XSIM measurements comparing tilt‐correction schemes before susceptibility calculation with TKD (D), iterative Tikhonov regularization (E), and linear weighted linear TV (F) methods are also in agreement with the unpadded results and x‐axis rotations (Figure 6, bottom row; Supporting Information Figure S1D–F) Figure S3. Numerical phantom results from rotations about the y = x‐axis with padded image volumes to ensure no parts of the original image volume were cut off during rotations. The XSIM measurements of QSM during the background field removal part of the pipeline for PDF (A), LBV (B), and V‐SHARP (C) agree with the unpadded results and rotations about the x‐axes and y‐axes. The XSIM [file MRM-89-1791-s001.docx]

**Supporting Information**


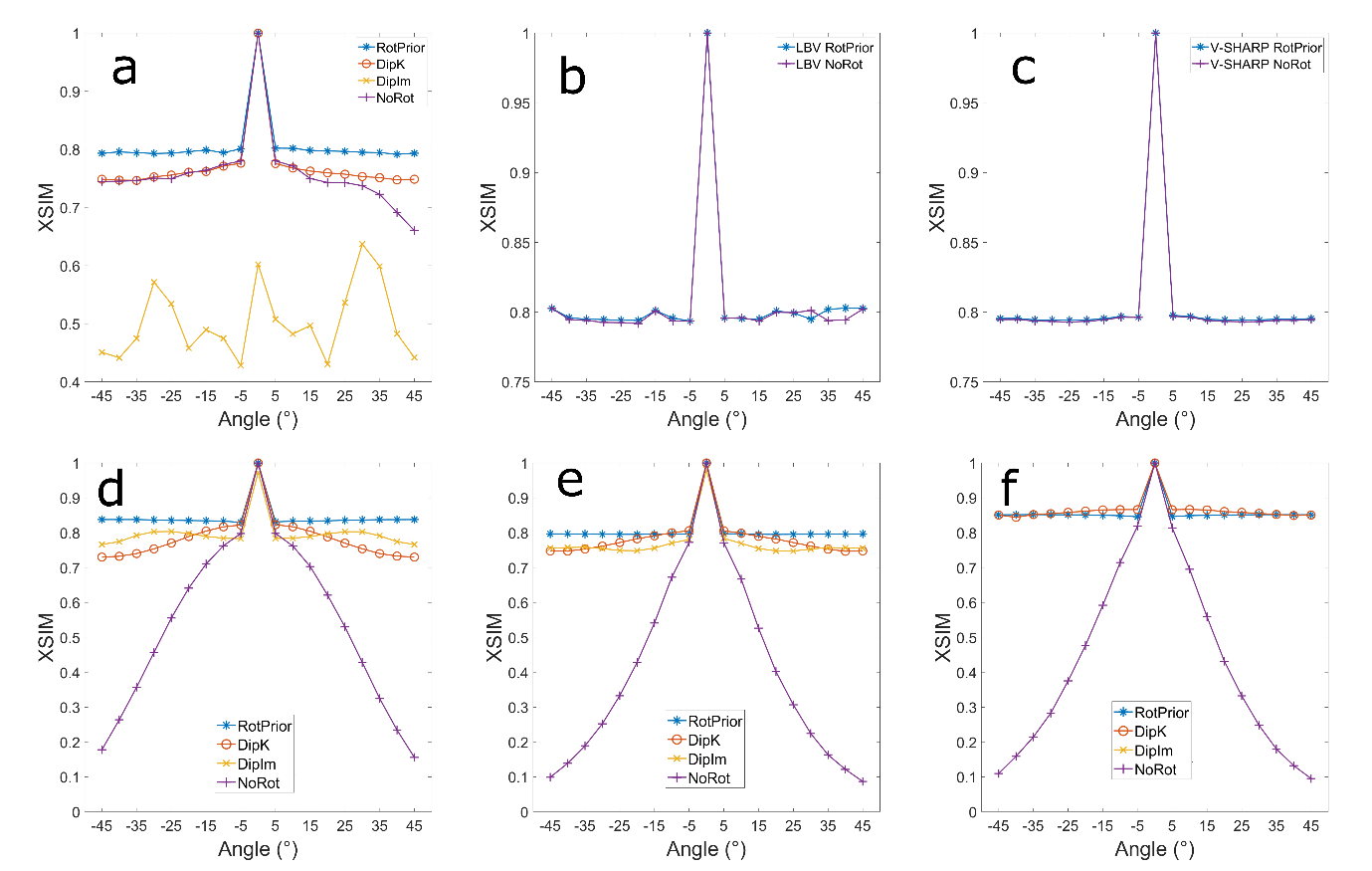


Supporting Information Figure S1: Results in the numerical phantom from oblique image volumes tilted about the x-axis. The volumes were padded to ensure no parts of the original image volume were cut off during rotations. XSIM measurements of QSM calculated with tilt corrections prior to background field removal with PDF (a), LBV (b) and V-SHARP (c) agree with unpadded results (Figure 5). XSIM measurements comparing tilt correction schemes prior to susceptibility calculation with TKD (d), iterative Tikhonov regularisation (e) and linear weighted linear TV (f) methods are also in agreement with unpadded results (Figure 6, bottom row).


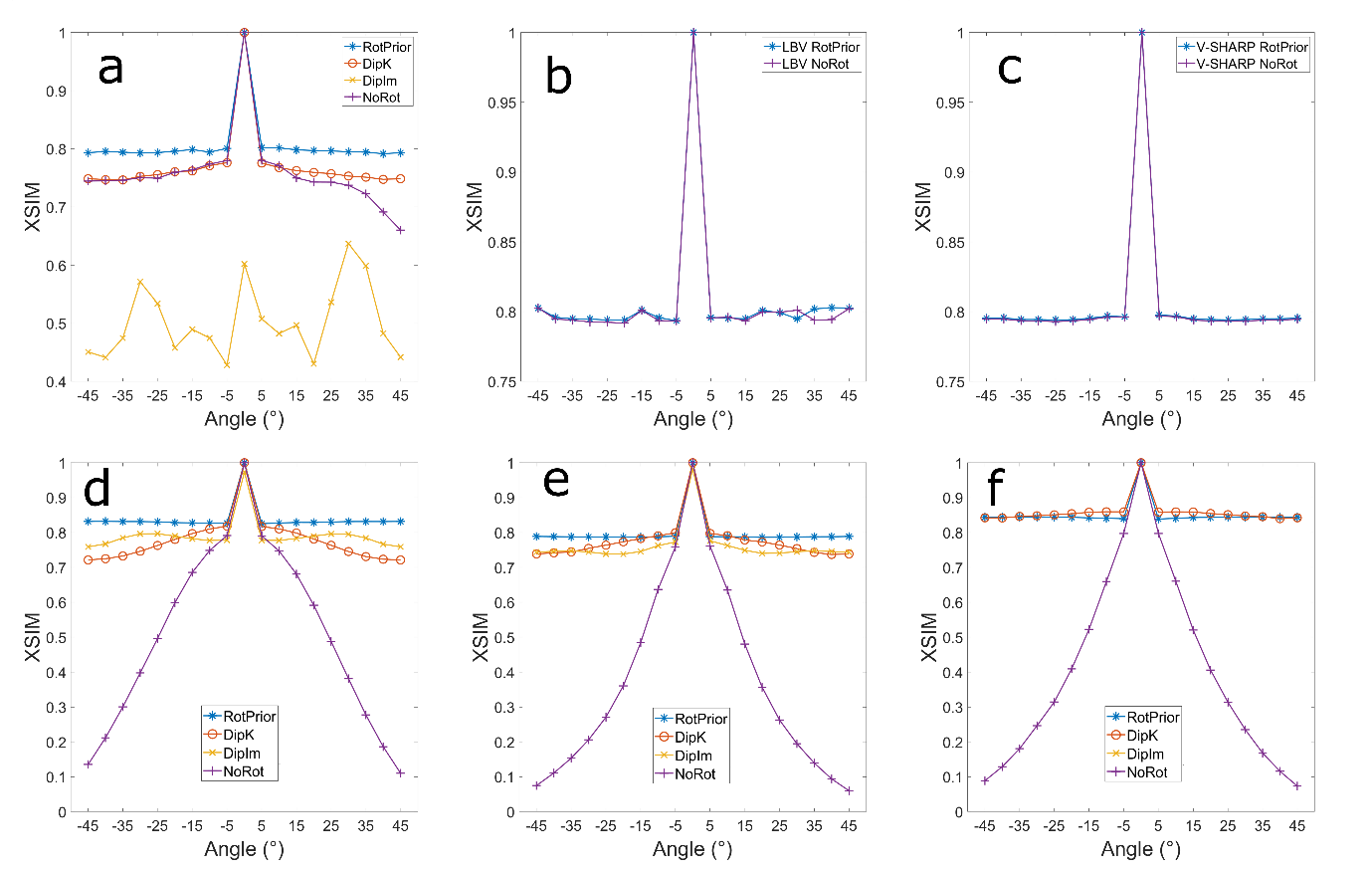


Supporting Information Figure S2: Results in the numerical phantom from oblique image volumes tilted about the y-axis with padded image volumes to ensure no parts of the original image volume were cut off during rotations. XSIM measurements of QSMs calculated with tilt corrections prior to background field removal with PDF (a), LBV (b) and V-SHARP (c) agree with unpadded results and rotations about the x-axis (Figure 5, Supporting Information Figure S1a,b,c). XSIM measurements comparing tilt correction schemes prior to susceptibility calculation with TKD (d), iterative Tikhonov regularisation (e) and linear weighted linear TV (f) methods are also in agreement with unpadded results and x-axis rotations (Figure 6, bottom row; Supporting Information Figure S1 d, e, f).


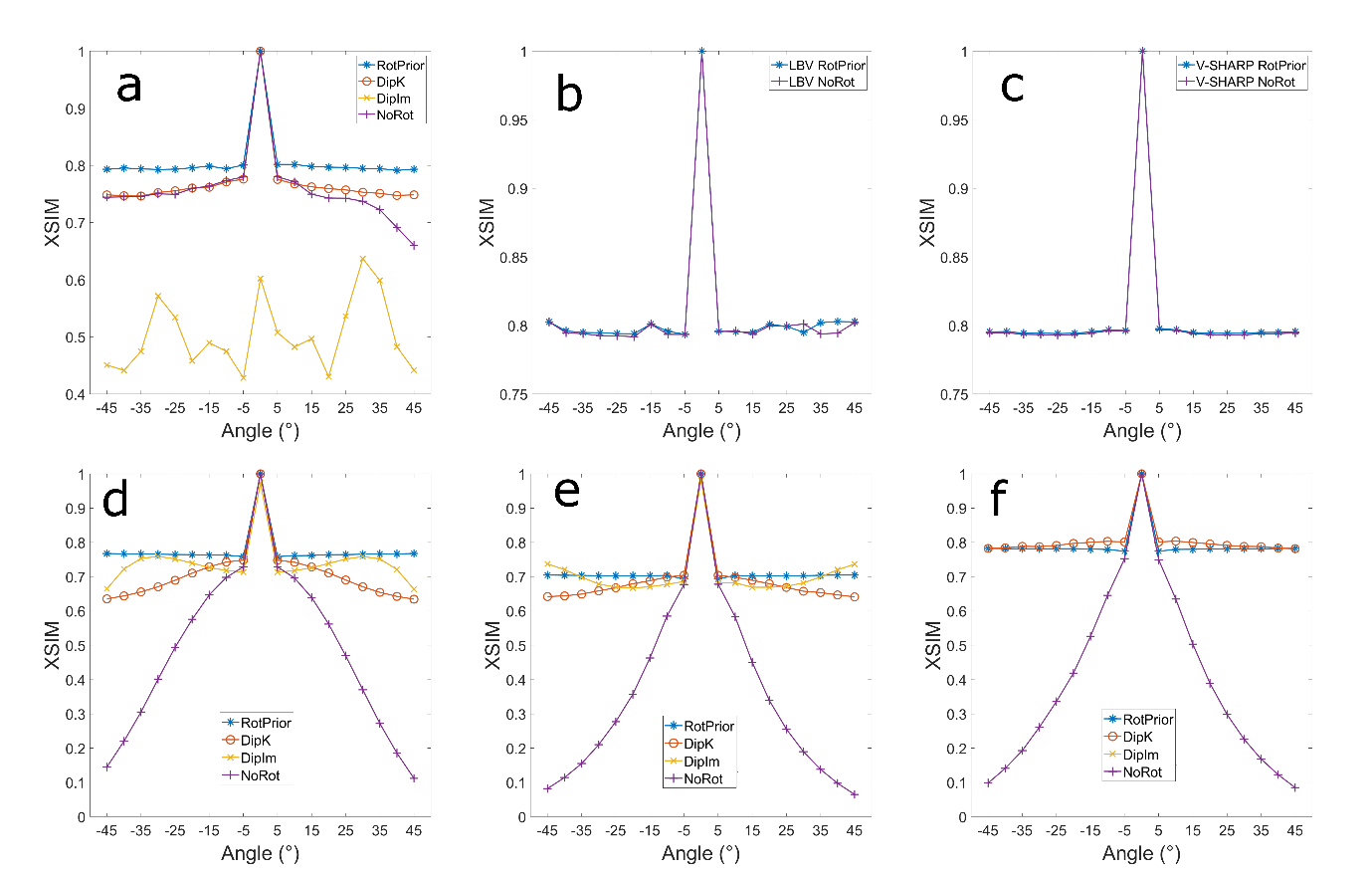


Supporting Information Figure S3: Numerical phantom results from rotations about the y=x axis with padded image volumes to ensure no parts of the original image volume was cut off during rotations. XSIM measurements of QSMs during the background field removal part of the pipeline for PDF (a), LBV (b) and V-SHARP (c) agree with unpadded results and rotations about the x and y-axes. XSIM measurements comparing susceptibility calculation methods TKD (d), iterative Tikhonov regularisation (e) and linear weighted linear TV (f) are also in agreement with unpadded results and x and y axis rotations (Figure 6, bottom row; Supporting Information Figures S1 and S2 d, e, f).


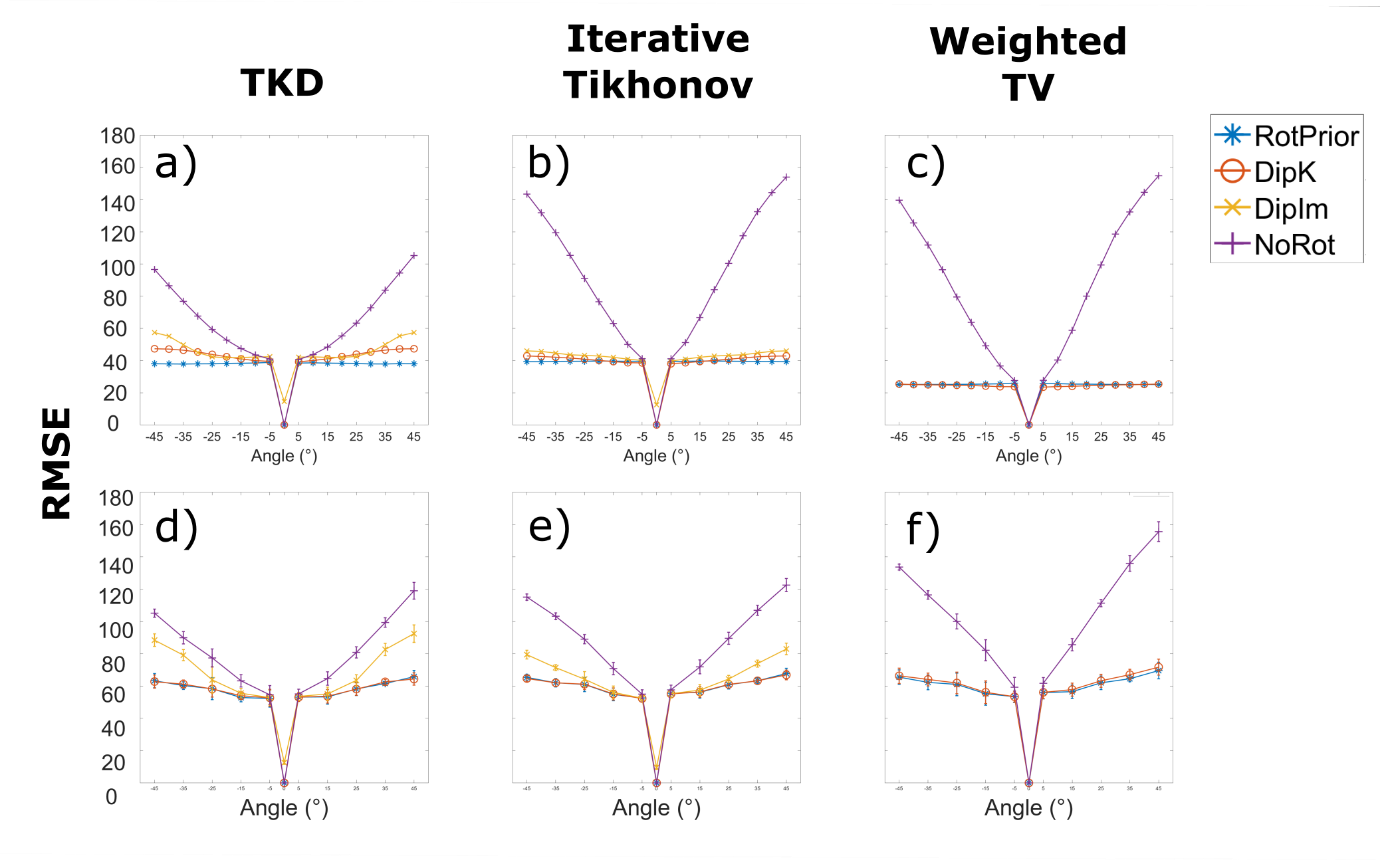


Supporting Information Figure S4: RMSE plots over all angles for all tilt correction schemes and all three susceptibility calculation methods in the numerical phantom (a-c) and averaged across all healthy volunteers (d-f). These results agree with the XSIM measurements found in Figures 6 and 9, for the numerical phantom and the in vivo results, respectively. Error bars (d-f) represent the standard deviation on the mean across all volunteers.


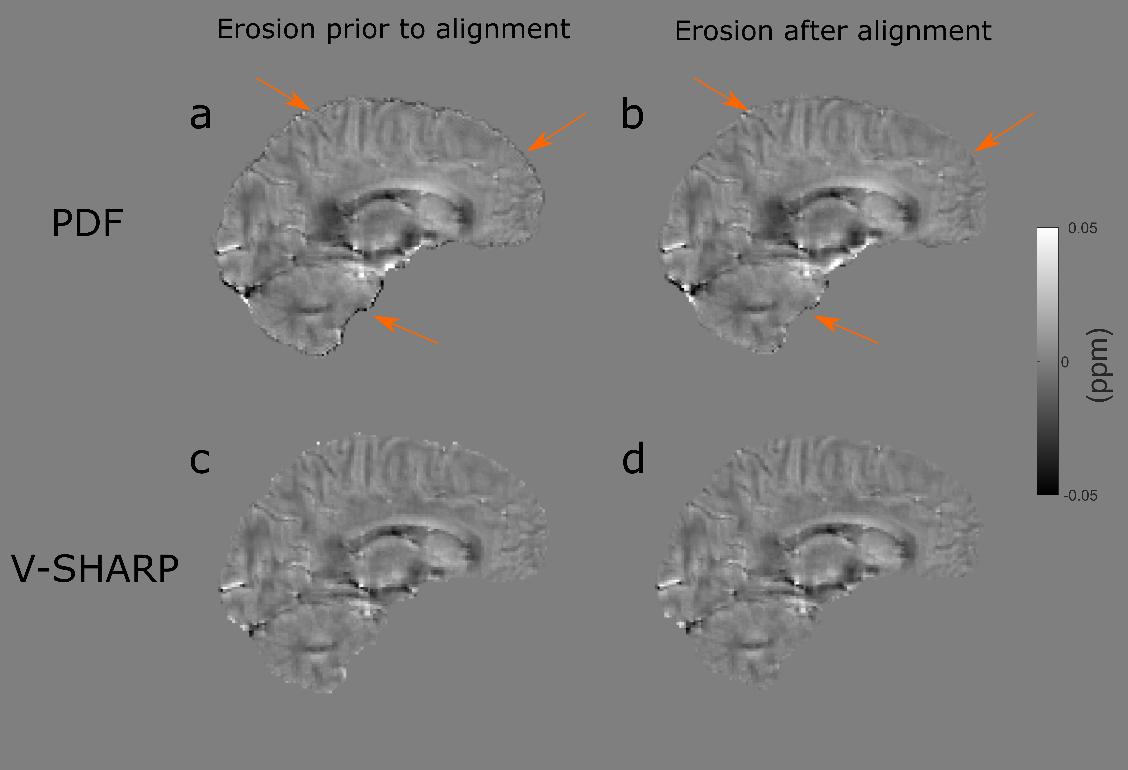


Supporting Information Figure S5: For improved PDF performance, the brain mask is typically eroded. If this erosion takes place prior to rotating the field map into alignment with ${\hat{\boldsymbol{B}}}_{\boldsymbol{0}}$ (a) compared to after (b), artefacts arise along the edges of the local field map following background field removal with PDF (a, orange arrows), increasing the RMSE and decreasing the XSIM. These artefacts do not arise when using V-SHARP (c: erosion before, d: erosion after).


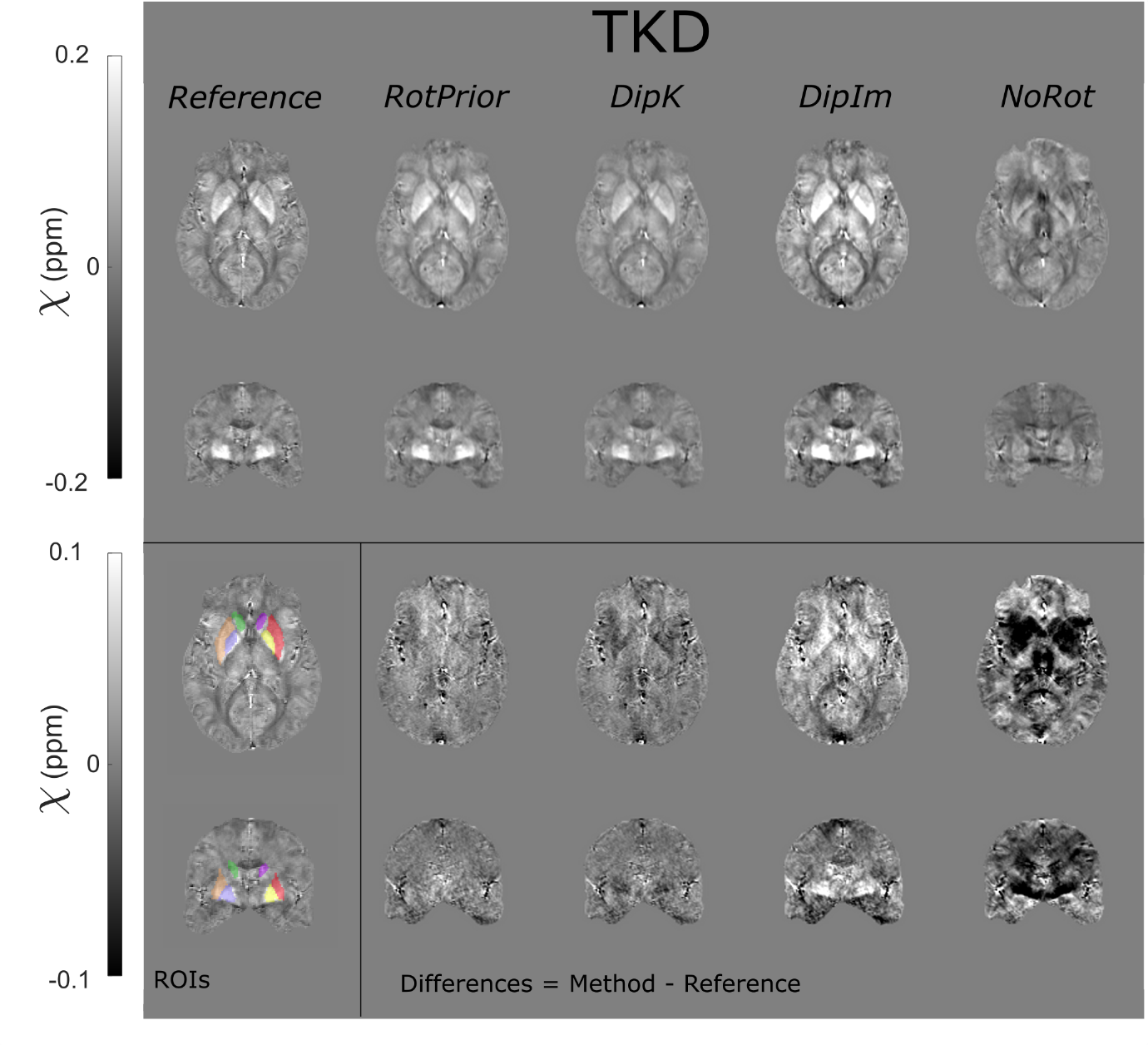


Supporting Information Figure S6: χ maps and difference images illustrating the effects of all tilt correction schemes on susceptibility calculation in vivo. An axial and a coronal slice are shown for a volume tilted at 45° and a reference (0°) volume with all χ maps calculated using Thresholded k-space (TKD) method. NoRot leads to the largest differences and image artefacts throughout the brain. The EVE ROIs used are shown (bottom left). These results are very similar to iterative Tikhonov and weighted linear TV susceptibility maps (Figure 10).


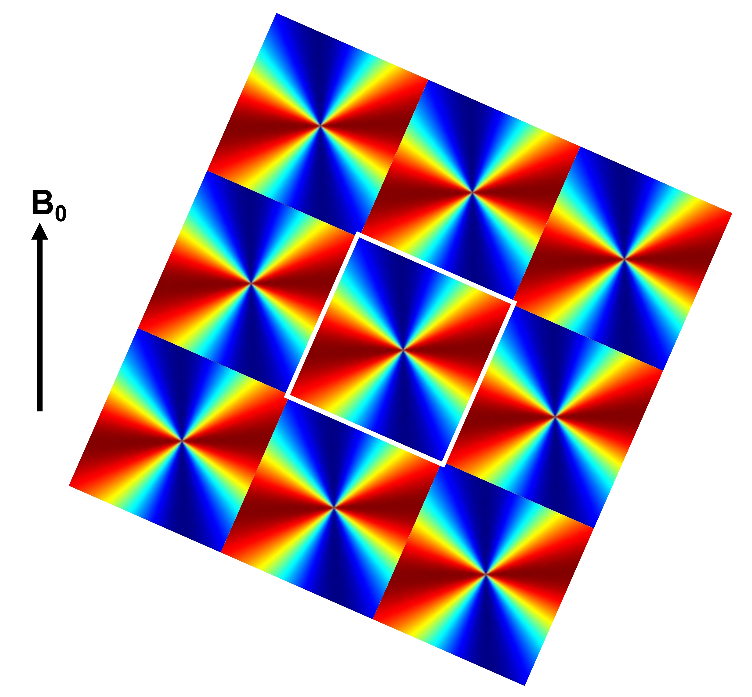


Supporting Information Figure S7: Oblique k-space magnetic dipole kernels laid side by side to illustrate the violations in circular continuity. These dipoles are used in the DipK correction method, which leads to striping artifacts due to the violations in circular continuity i.e. discontinuities at the boundaries of the rotated k-space dipoles (white square and arrows).


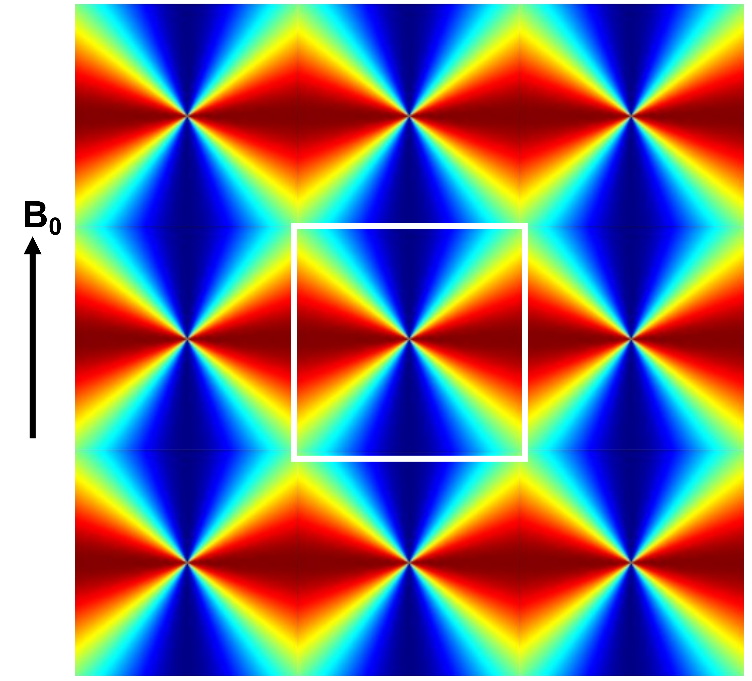


Supporting Information Figure S8: Non-oblique k-space magnetic dipole kernels laid side by side to illustrate circular continuity. When there is no oblique acquisition then there are no violations in circular continuity i.e. identical values and no discontinuities at the boundaries of the k-space dipoles (white square).
